# Structural and dynamical characteristics of flow units in metallic glasses

**DOI:** 10.1038/s41598-017-11139-7

**Published:** 2017-09-14

**Authors:** S. T. Liu, F. X. Li, M. Z. Li, W. H. Wang

**Affiliations:** 1 Department of Mechanics and Engineering Science, LTCS, and CAPT, College of Engineering, Peking University, Beijing, 100871 China; 2 Institute of Physics, Chinese Academy of Sciences, Beijing, 100190 China; 3 Department of Physics, Beijing Key Laboratory of Opto-electronic Functional Materials & Micro-nano Devices, Renmin University of China, Beijing, 100872 China

## Abstract

The metallic glasses (MGs) are conjectured to be heterogeneous—their microscopic structures are embedded with localized, soft and loosely packed atomic regions, which are termed as flow units (FUs). Detailed knowledges on the structure and dynamical features of FUs are essential for understanding the plasticity of MGs. In our study, by performing dynamical tests on MGs in molecular dynamics simulations, we show that mechanical hysteretic loops are formed in the strain-stress curves due to the undergoing plastic events. By analyzing the activated times of each atom in different dynamical tests, we map the exact locations of FUs and the distribution of their activation probability in the initial structure of MGs. More importantly, we demonstrate that the FUs are indeed liquid-like according to the Lindemann criterion of melting.

## Introduction

Because metallic glasses (MGs) do not possess slip systems or lattice dislocations as in crystalline materials^1–4^, the structure origin and their dynamical response for deformation in MGs is still elusive and have drawn a lot attentions^5–13^. Recent studies suggest that the structures of MGs are heterogeneous^14–18^, consisting of densely and loosely packed regions^17–21^. The latter is referred as flow units (FUs), which are believed to be the basic units of deformation, plasticity and viscoelasticity in MGs^23, 24^. However, it is much more desirable to know where the FUs locate in MGs and what structural and dynamical features they have, which are essential for understanding the plastic events from the initial structures in MGs. A few experiments prove that before the yield of MGs, the nucleation of the shear bands have already formed and taken effect on the mechanical response^21–26^. A typical example is shown by the mechanical hysteretic loops in the dynamical tests, indicating the plastic behavior of MGs in elastic regime^24–26^. The dynamical test conducted in elastic regime offers an effective way to stimulate the FUs and explore their mechanical response, dynamical behavior and spatial distribution without damaging the whole systems.

It has been conjectured that the FUs are loosely packed, liquid -like and embedded in the densely-packed regions, that are solid-like and form the skeleton of the MGs^22, 24^. However, no clear evidence has been revealed. The dynamics of both the FUs and solid-like regions, including the excitations and the percolations of FUs, are the fundamental processes that determine the mechanical property of MGs. However, few works have been devoted to exploring the vibration state and the related physical state of the two regions. To rationalize this hypothesis that MGs are mixture of liquid-like FUs and solid-like matrix, it is highly desirable to identify the fluidity of the heterogeneous structures in MGs.

In this study, we carry out multiple dynamical tests with different maximum loading stresses in the apparent elastic regime on a model CuZr MG system via molecular dynamics simulations. The mechanical hysteretic loops are observed in strain-stress curves which indicate the activation of FUs in these processes. Whether an atom is activated or not is represented by the changes in the number of its nearest neighbors. By mapping the activated times of atoms in the multiple dynamical tests onto the initial configuration, the exact locations of FUs and the heterogeneous feature of MG structures are unambiguously revealed. The FUs are loosely packed and activated in dynamical tests with a broad activation probability distribution. Analyses on the vibration spectrum show that the atoms in the FUs prefer to participate more in the low-frequency vibrational modes, implying the soft characteristic of the FUs in MGs. Furthermore, the dynamics and liquid-like feature of FUs in MGs at room temperature are quantitatively characterized in terms of the Lindemann criterion of melting as well as the extensive trajectories of atoms in FUs.

## Model and Method

Classical molecular dynamics (MD) simulations were performed for the model system of Cu_64.5_Zr_35.5_ metallic alloy with the interatomic interactions described by realistic embedded-atom method potential^27^. The system containing 40,000 atoms in a cubic with periodic boundary conditions in three dimensions was fully relaxed and equilibrated at 2000 K, and then quenched down to 300 K with a cooling rate of 1 × 10^12^ K/s. Subsequently, the quenched sample was relaxed for 2 ns at 300 K. All MD simulations were performed in isothermal-isobaric (NPT) ensemble with pressure adjusted to be zero and the MD step is 2 fs. To identify FUs in the MG sample, dynamical tests with different maximum loading stress σ_max_ were performed in the apparent elastic regime. In specific, the uniaxial tensile stress was applied to the sample in z direction with a constant loading rate of 2 × 10^9^ GPa/s until a maximum loading stress σ_max_ was reached, and then the applied tensile stress was fully unloaded with the same rate. The dynamical tests were repeated for the same initial configuration but with different maximum loading stresses σ_max_. In our study 22 dynamical tests were performed to examine the response of FUs in the sample.

## Result and Discussion

### Hysteretic loops in strain-stress curves

**Figure 1:**
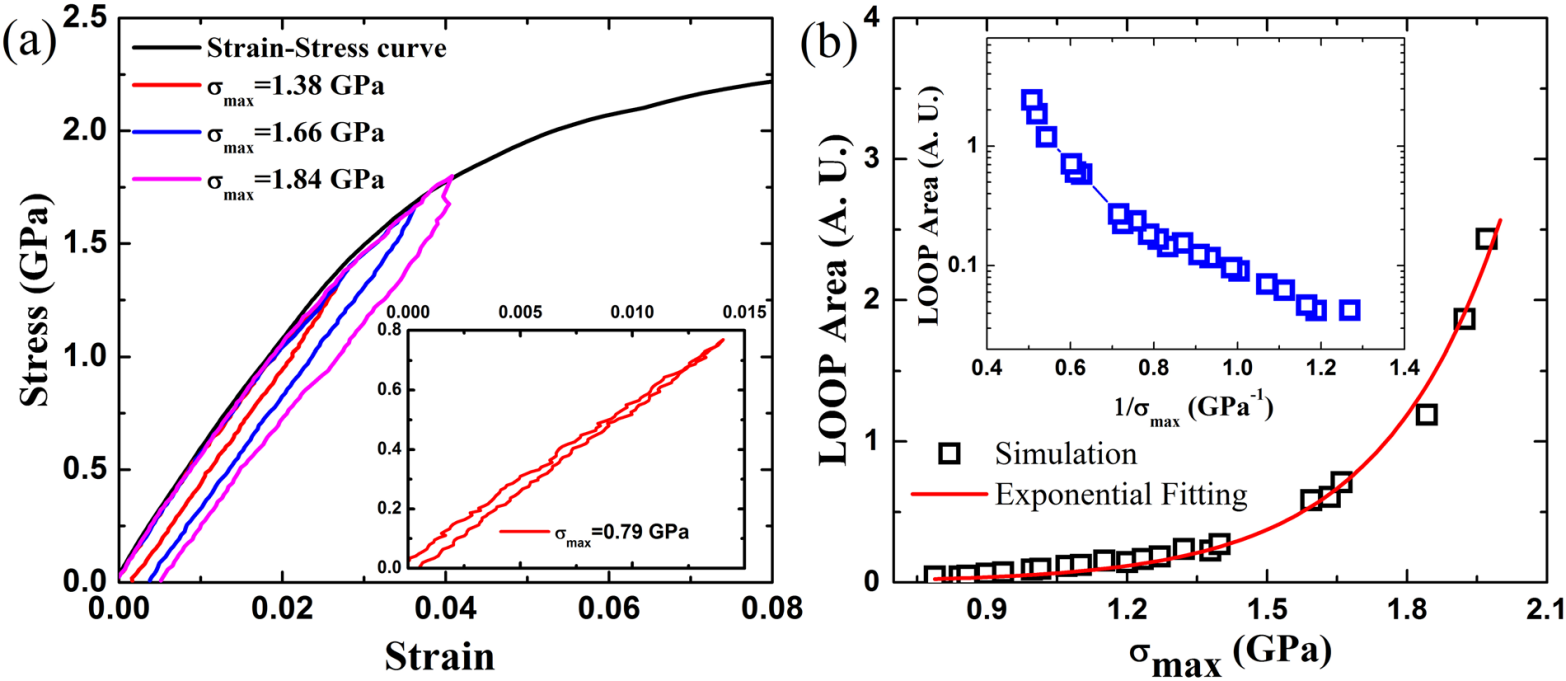
(**a**) Strain-stress curves in dynamical tests with different maximum loading stresses σ_max_ in apparent elastic regime. The inset shows the hysteretic loop in the case of σ_max_ = 0.79 GPa. (**b**) Dependence of the areas of the hysteretic loops on the maximum loading stresses σ_max_ in dynamic tests. Red curve is exponential fitting. The inset shows the semi-log plot of the dependence of the loop areas on 1/σ_max_.

Figure 1(a) shows the strain-stress curves in dynamical tests with different σ_max_. The strain-stress curve in the tensile loading up to plastic regime is also presented for comparison. The yield stress is estimated to be about 2.10 GPa and all the dynamical tests applied to the sample are in the apparent elastic regime. As shown in Fig. 1(a), the strain-stress curves in the dynamical tests exhibit mechanical hysteretic loops, consistent with the experimental observations^24–26^. The mechanical hysteretic loops in strain-stress curves indicate that the MG samples have experienced plastic deformations in the apparent elastic regime. The plasticity even occurs in the case of σ_max_ = 0.79 GPa as shown in the inset in Fig. 1(a), which is far below the yield stress.

From structural perspective, the plastic deformation in apparent elastic regime of the MGs results from the irreversible atomic rearrangements^24–26^. The regions experiencing irreversible rearrangements in dynamical tests are referred as FUs. The irreversible rearrangements, namely the activation of FUs, result in the plastic deformation in the elastic regime and mechanical hysteresis in the dynamical tests. As σ_max_ increases, the areas of the hysteretic loops increase as shown in Fig. 1(a), implying that more FUs in MGs are activated. Figure 1(b) shows that the loop area grows almost exponentially with increasing σ_max_ and can be well fitted by an exponential form.

To further confirm the activation behavior of FUs in MGs in dynamical tests, we examine the dependence of the loop area on 1/σ_max_ to obtain the activation energy information. The inset in Fig. 1(b) shows the semi-log plot of loop area v.s. 1/σ_max_ and the slope of the curve is related to the activation energy. The results indicate that the activation energies of FUs are quite different and have a distribution in MGs: some FUs have small activation energies which can be easily activated under very small stress, while others can only be activated under large loading stress. The distribution of the activation energy due to the dynamic and structural heterogeneities in MGs^19^ is consistent with that obtained in experiments^28^. Note that according to the previous studies^29, 30^, the hysteretic loops in dynamical tests could be related to the anelasticity. However, it is not the case in our study, since the time scale in the unloading process (300~1000 ps) is much longer than that used for the anelastic recovery in previous studies (10~30 ps)^29^, so that the contribution of anelasticity to hysteretic loops in our dynamical tests is negligible, and the hysteretic loops mainly result from the local plastic events of FUs.

### Quantify and identify the FUs

To quantify the atomic rearrangements and identify the FUs in MGs, we measured the number of changes in the nearest neighbors of each atom Δ*NN* before stress loading and after unloading, which has been used to identify slow particles over a time difference^31^. The nearest neighbors of atoms are determined by Voronoi tessellation method^13^. Small values of Δ*NN* indicate that the atoms deform elastically during certain dynamical test. After fully unloaded, the initial configurations of these atoms are almost recovered. In contrast, larger values of Δ*NN* indicate that the atomic rearrangements are irreversible and the atoms experience plastic deformation. Thus, the atoms with large Δ*NN* are the representatives of FUs in MGs, and we may characterize the atoms with large Δ*NN* to get information on the atomic structural and dynamical features in FUs.

We also analyze the motion of each atom between the initial and final configurations in the dynamical tests to examine the mobility of atoms. Here the mobility of atom *i* is defined as ${\rm{\Delta }}{r}_{i}^{2}={|{{\bf{r}}}_{i}(1)-{{\bf{r}}}_{i}(0)|}^{2}$ ^13^, where ${{\bf{r}}}_{i}()$ denotes the atomic position of atom *i*. The indexes 0 and 1 denote the atomic configurations before loading and after unloading in the dynamical test, respectively. Therefore, the atomic mobility defined here can be used to measure the displacement an atom moves in a dynamical test.

**Figure 2:**
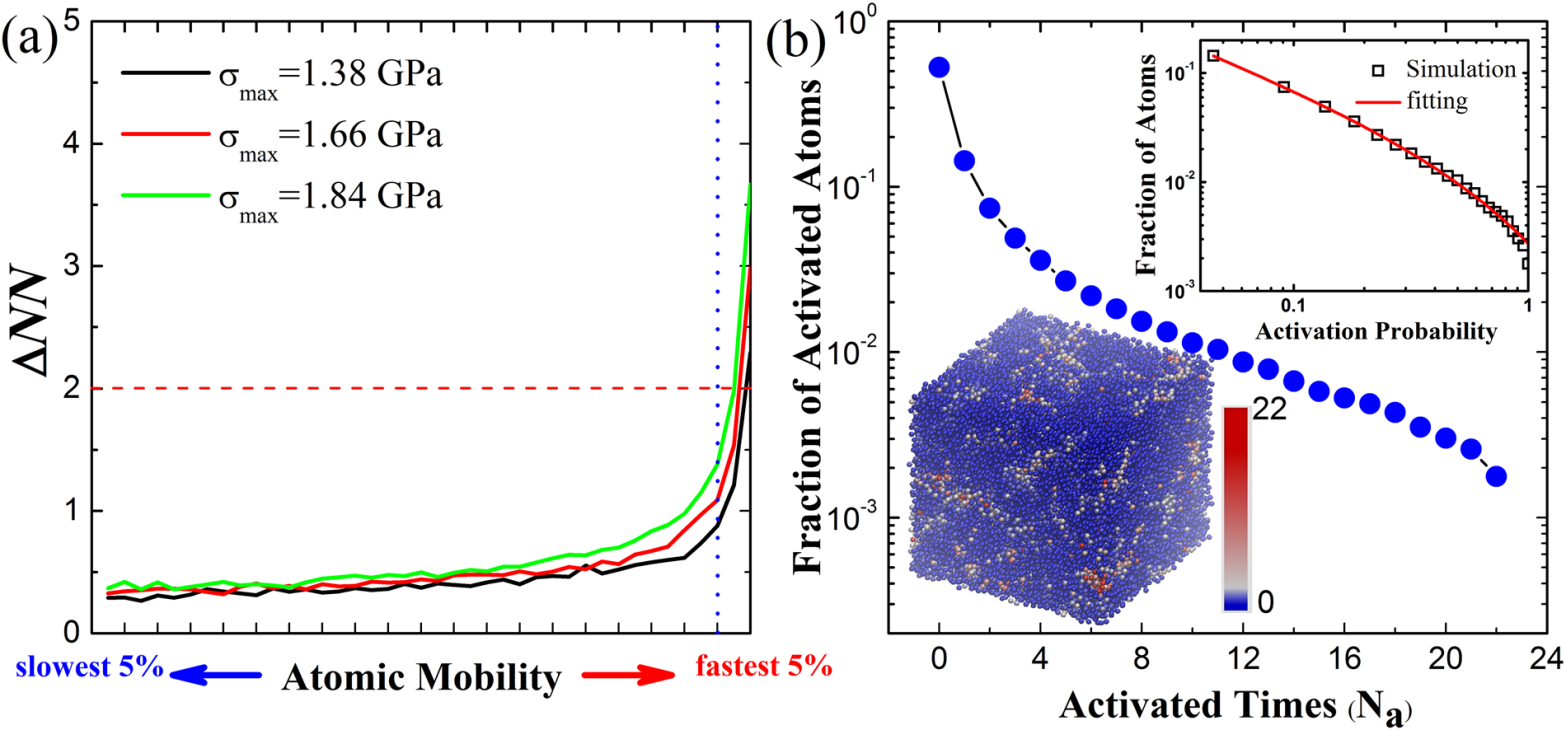
(**a**) Correlation between the number of changes in the nearest neighbors of atoms Δ*NN* and the atomic mobility Δ*r* ^2^ before and after loading. The curves are actually histograms with 5% atoms per bin. Sharp Δ*NN* increase occurs for the fastest 5% atoms marked by the vertical dotted line. The atoms with Δ*NN* ≥ 2 marked by the red dashed lineare assumed to be activated. (**b**) The fraction distribution of atoms with respected to their activated times *N*_a_ among 22 dynamic tests in MGs. The upper inset: the log-log plot of the fraction of atoms vs. activation probability; The lower inset: the atomic configuration colored with the activated times of each individual atoms.

Figure 2(a) shows the correlation between Δ*NN* and Δ*r* ^2^ of atoms in different dynamical tests. The atoms are sorted in terms of their atomic mobility from low to high and divided into 20 groups, each containing 5% (2000) of the total atoms. In each group, the average Δ*NN* is calculated and plotted as curves in Fig. 2(a). It is clear that the atoms with small values of Δ*NN* are immobile, while the atoms with large Δ*NN* are more mobile. As atomic mobility increases, the values of Δ*NN* increase abruptly. These atoms in FUs are much more mobile and experience irreversible rearrangements under stress. It is also shown that for most atoms the values of Δ*NN* are less than 2 and immobile, so that the atomic configurations of these atoms are almost unchanged in the loading-unloading dynamical tests, and mainly experience elastic deformation. These atoms form the so-called solid-like elastic matrix in MGs. It is clearly seen that only a small fraction of atoms with large Δ*NN* experiences irreversible rearrangements and makes contribution to the mechanical hysteretic loops in dynamical tests. According to Fig. 2(a) in our study the atoms with Δ*NN* ≥ 2 are assumed to be activated atoms in FUs during the dynamical tests. We also tested the different choice of Δ*NN*, for example, Δ*NN* ≥ 3 was used to identify the FUs in the samples, which does not qualitatively violate the results and conclusions obtained below.

To reveal the intrinsic FU structure in MGs where the irreversible atomic rearrangement is prone to occur, we statistically count the times *N*_a_ of each atom activated (Δ*NN* ≥ 2) among the 22 dynamical tests. If an atom is activated in all 22 dynamical tests, we have *N*_a_ = 22, while *N*_a_ = 0 means that an atom is never activated in these tests. The activation probability of each individual atom is determined as *P* = *N*_a_/*N*_test_ (*N*_test_ = 22) and their distribution is shown in Fig. 2(b). As can be seen, a large part of atoms (about 50%) in the samples are almost stable against loading stress and never activated in these dynamical tests, which corresponds to the solid-like elastic matrix in MGs. It is also found that a small portion of atoms (~0.17% in our case) are always activated in all dynamical tests. These atoms correspond to the most active cores of the FUs.

The distribution of activation probability of atoms can be well-fitted by a power-law form with exponential cutoff, *f*(*P*)*~P* ^−*α*^ *e* ^−*P/β*^ as shown in the upper right inset in Fig. 2(b). Here *f* represents the fraction of atoms; *α* and *β* are fitting parameters and fitted to be 0.85 and 0.61, respectively. The fitted parameters imply that if the activation probability is larger than 0.61, the regions with the corresponding atoms can be regarded as FUs in MGs, which is responsible for the stress-induced irreversible atomic rearrangements in MGs in elastic deformation regime and consistent with the experimental measurements^22–27^. The spatial distribution of the FUs and elastic matrix can be exhibited by mapping the activated times *N*_a_ of all the atoms into the initial configuration as shown in the lower left inset in Fig. 2(b). The red and blue represent large and small *N*_a_ of atoms, respectively. It is clearly seen that the atoms with larger *N*_a_ are highly localized regions embedded in the elastic matrix, confirming the structural heterogeneity^19, 20, 22^. The FUs in MGs are almost enclosed by the elastic matrix, roughly forming the so-called core-shell structure as observed in experiments^24^. These findings also indicate that the structural characteristic of FUs could be used to evaluate where the irreversible atomic rearrangements would occur from the initial structures of MGs and with what probability. This is comparable to the local Deybe-Waller factor which has been used to predict the spatial distribution of the long-time dynamic propensities^32^. In our work, explicit initial structural information is provided.

### Independence of different loops

**Figure 3:**
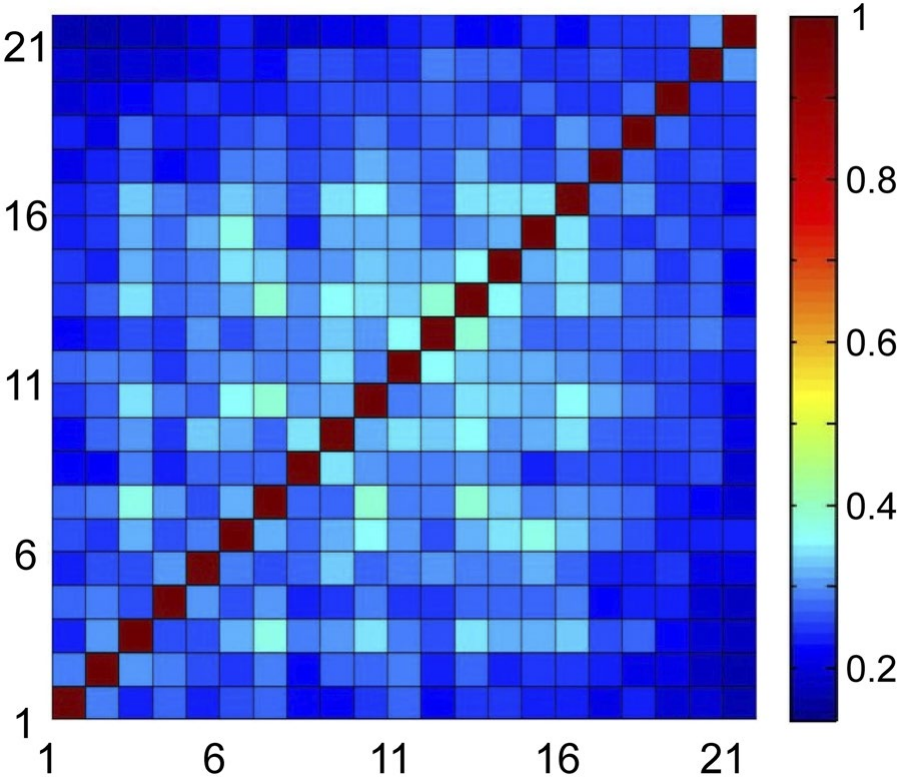
The correlation matrixes for the activation distributions of 22 different dynamic tests. The values of the sub-, super-diagonal elements (m = n − 1 and m = n + 1) and other off-diagonal elements (m > n − 1 and m < n − 1) persist around 0.3~0.4. The high correlation of the spatial distributions during 22 different dynamic tests can be clearly seen.

We show below that the 22 dynamical tests are found to be independent of each other, which further demonstrates the FUs are intrinsic in MGs. In Fig. 3, the correlation matrix for the activation distributions of the different dynamic tests is presented. Here the elements of the correlation matrix are defined as:

1 $${C}_{mn}={\rm{cov}}({p}_{i}^{m},{p}_{i}^{n})/\sqrt{{\rm{cov}}({p}_{i}^{m},{p}_{i}^{m}){\rm{cov}}({p}_{i}^{n},{p}_{i}^{n})}$$

where ${p}_{i}^{m}$ denotes whether the atom *i* is activated or not in the dynamic test *m*: ${p}_{i}^{m}=1$ for Δ*NN* > 2 and ${p}_{i}^{m}=0$ for Δ*NN* < 2. The cov() is the ordinary covariant function. The elements *C*_*mn*_ show the correlations of the activation distributions *p*_*i*_ for the two dynamic tests *m* and *n*. In the matrix, the tests are sorted by their applied maximum loadings, which also show the temporal evolution of the system.

As shown in Fig. 3, the diagonal elements of the matrix represent the self-correlations of the tests. The values of the elements are all 1, showing maximum correlations. The sub- and super-diagonal elements (*m* = *n* − 1 and *m* = *n* + 1) show the correlations between two dynamic tests with close maximum loadings. As can be seen, the values of these elements sharply decrease to about 0.3. The values of other off-diagonal elements (*m* > *n* − 1 and *m* < *n* − 1), that denote correlations between two dynamic tests with larger difference in the maximum loadings, persist around 0.3~0.4. The sharp decreasing reveals the poor temporal correlations of the dynamic tests, which imply the activations in the MGs are fast processes. In spite of the poor temporal correlations, considering that the random distributions give value of zero for the correlations, the spatial distributions for the activations are still highly correlated (shown by the value of off-diagonal elements) during the whole elastic loading process. These high correlations are not due to the adjacency in time sequence, but owing to the tendency of the activations occurring on the intrinsic FU structure in the MGs. For elements with large *m* or *n*, the correlations begin to drop because of the increase in the spatial randomness of the activations due to percolations. This matrix offers a clear scenario occurring in the MGs: the activations are created and annihilated rapidly during the loading, but the activations are prone to happen on the intrinsic FU structures in the MGs.

### Structural characteristics of the FUs

**Figure 4:**
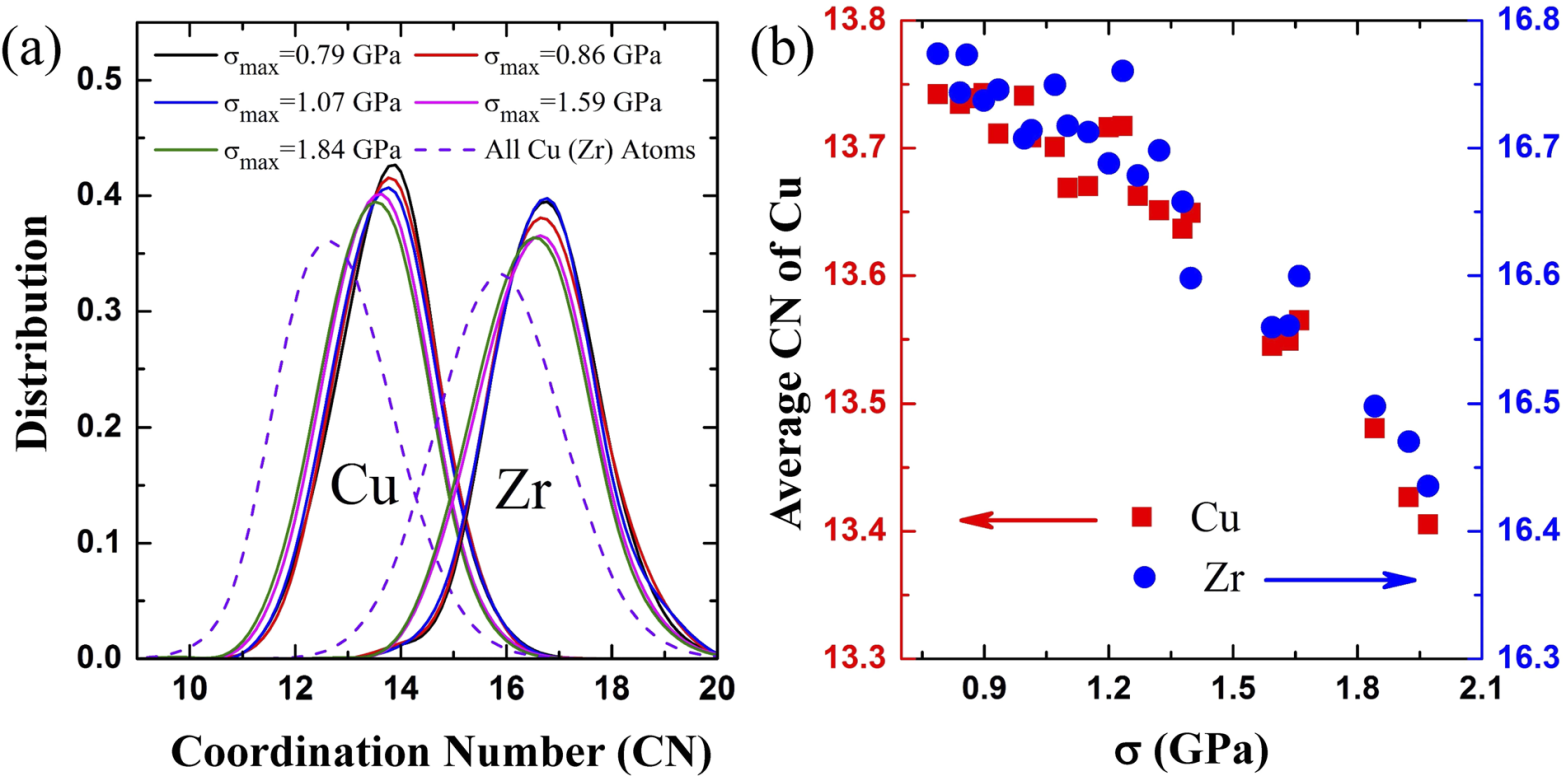
(**a**) The distribution of coordination numbers (CN) of Cu and Zr atoms with Δ*NN* ≥ 2 and those in whole sample in different dynamical tests. (**b**) The change of average CN of Cu (12.8) and Zr (15.9) atoms with Δ*NN* ≥ 2 with σ_max_ increasing.

We simply analyze the coordination number (CN) of the activated atoms (Δ*NN* ≥ 2) in different dynamical tests to characterize the local atomic packing of FUs in MGs. Figure 4(a) shows the CN distributions of activated Cu and Zr atoms in different dynamical tests and the comparison with that of the whole sample. All the CN distributions are Gaussian-like, while the CN of the selected atoms is distributed in the range of larger CN, which indicates that the local atomic packing of the atoms in FUs is more loosely than the average. This result is also consistent with previous studies, which found that unstable local structures of loosely packed atoms have higher tendency for deformation^33–38^. It is interesting to find that the CN distribution of the selected atoms in the dynamical test of the smallest σ_max_ locates the farthest away from the average distribution, and then moves toward the average distribution as σ_max_ increases. This tendency is illustrated more clearly by the change of average CNs in FUs (Δ*NN* ≥ 2) with σ_max_ increasing in Fig. 4(b). For the smallest σ_max_ (0.79 GPa), the average CN values for Cu and Zr in FUs (Δ*NN* ≥ 2) are 13.75 and 16.77 respectively, much larger than the average CN values of 12.8 and 15.9 in the whole sample. As σ_max_ increases, the average CN of both Cu and Zr atoms with Δ*NN* ≥ 2 decreases.

The evolution of CN distribution of the activated atoms with increasing σ_max_ uncovers the underlying activation process of FUs. At the early stage, when small stress is applied, only the most loosely packed FUs in MGs are initially activated so that the irreversible atomic rearrangements are localized in the most loosely packed regions, leading to the plastic deformation in elastic regime. As σ_max_ increases, the activations began to occur in less loosely packed regions. When σ_max_ approaches the yield stress, the activations occur more randomly in the whole sample, which start to percolate and form the shear bands, rendering the macroscopic mechanical plasticity in MGs^39–42^.

**Figure 5:**
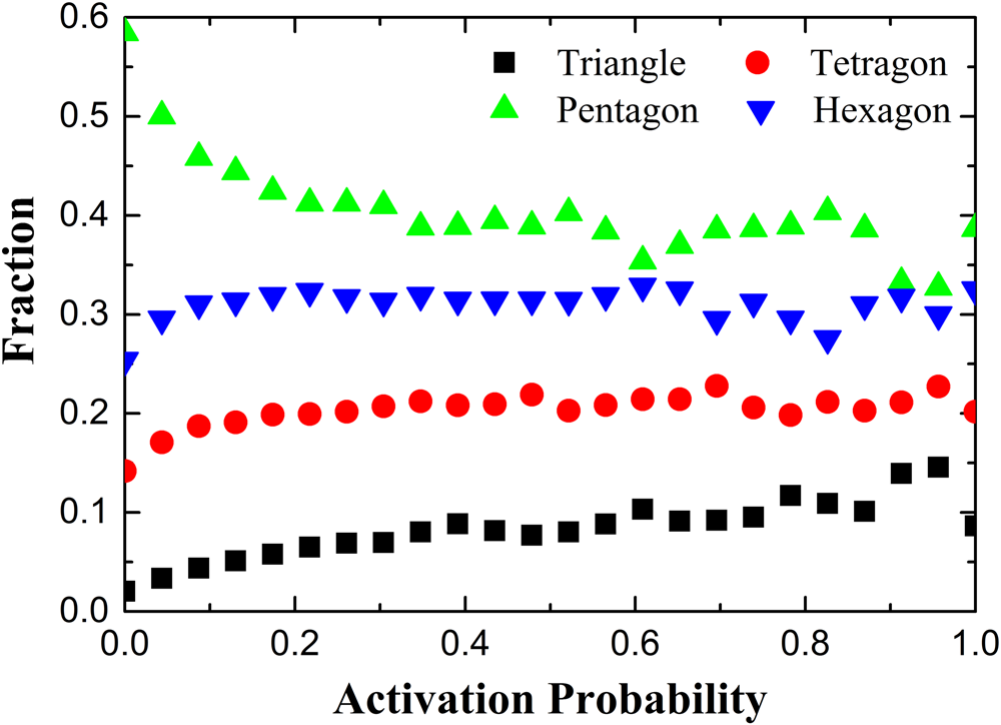
Change of the fraction of triangle, tetragon, pentagon and hexagon in atoms with activation probability. The open star shows the total fraction of triangle, tetragon and hexagon.

We also examine the local structure characteristic of FUs by Voronoi analysis, which shows that the FUs are packed with much less degree of local five-fold symmetry compared to the elastic matrix and further demonstrates the property of FUs and elastic matrix from structural perspective, as shown in Fig. 5. The Voronoi polygons such as triangles, tetragons, pentagons, and hexagons represent the atomic packing configurations formed by the central atom and a part of its nearest-neighbor atoms. The pentagon indicates that the corresponding atomic packing has local five-fold symmetry. In contrast, triangle, tetragon, and hexagon indicate that the atomic packings have local crystalline symmetry. Therefore, we compare the fraction of pentagon with other polygons and examine the change of the fraction with activation probability. As shown in Fig. 5, the fraction of pentagon decreases as the activation probability increases, while the fraction of the triangle, tetragon and hexagon increases. The total fraction of the triangle, tetragon and hexagon is also presented. It is clear that the fraction of pentagon is about 20% higher than other polygons in the atoms with the lowest activation probability. In contrast, in the atoms with the highest activation probability, the fraction of polygons with local crystalline symmetry is more than 20% higher than pentagon. Thus, the atoms in FUs are much more likely to pack with local crystalline symmetry, while more local five-fold symmetry is populated in solid-like elastic matrix which can resist the mechanical deformation.

### Dynamical properties of the FUs

**Figure 6:**
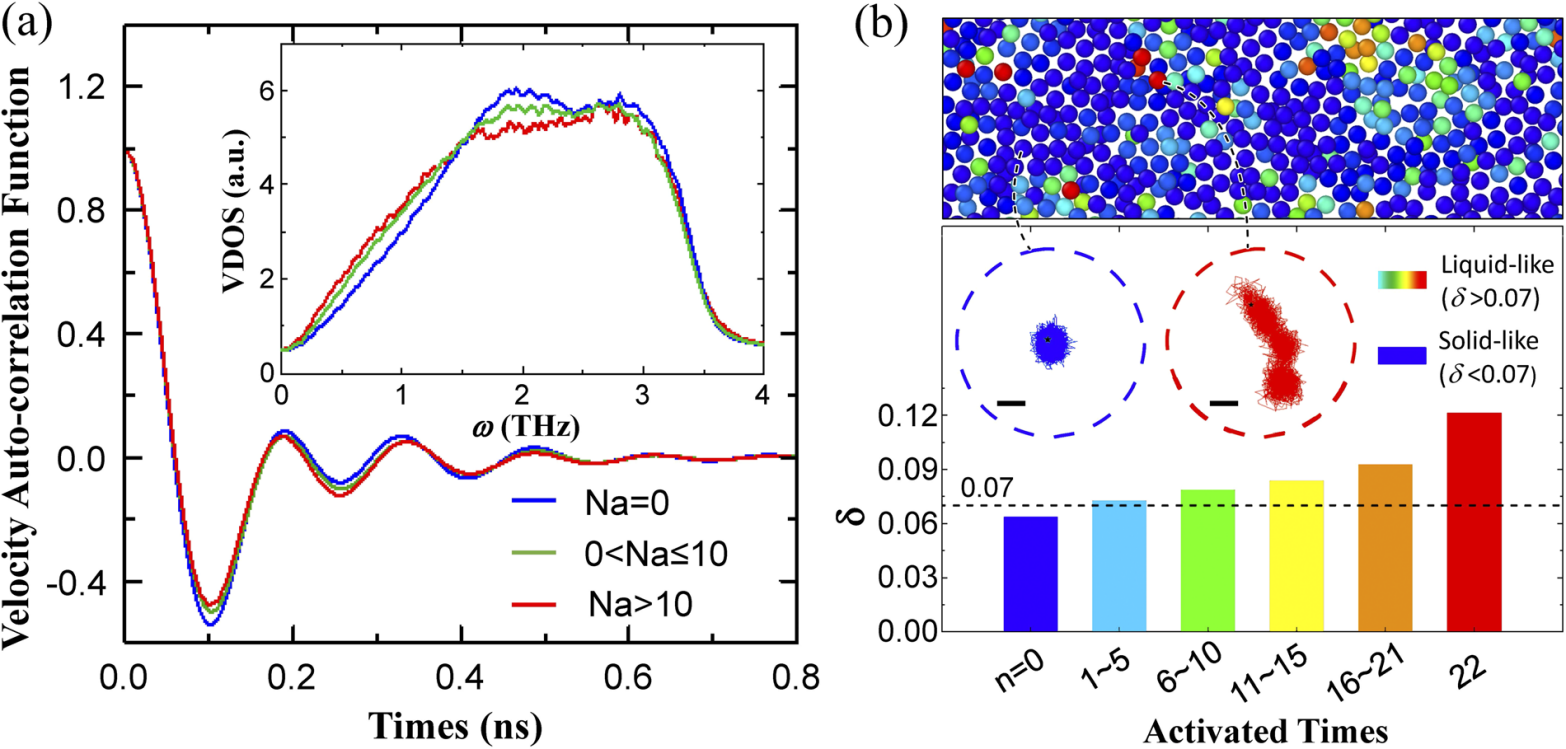
(**a**) VAFs for three groups of Cu atoms. Inset: the vibration density of states obtained by Fourier transform of VAFs. (**b**) Bottom panel: the Lindemann parameter of Cu atoms with different activated times. The inset: the typical trajectories of Cu atoms with *N*_a_ = 0 and *N*_a_ = 22, respectively. Scale bar: 0.5 Ǻ. Top panel: atomic configuration taken from the initial structure, illustrating the liquid-like FUs embedded in solid-like elastic matrix in MGs revealed by our analysis.

To get dynamical characteristics of the FUs in MGs, isothermal relaxation was performed in NVT ensemble for the initial configuration at 300 K for 2 ns via MD simulations, and the vibration power spectrum and long-time dynamics of atoms with different activated times *N*_a_ are analyzed. To obtained the vibration spectrum, the velocity auto-correlation function (VAF) ($v(0)\cdot v(t)$) of the atoms with different activated times is calculated. To avoid the influence introduced by the component difference, only Cu atoms are considered and divided into three groups according to the activated times (*N*_a_ = 0, 0 < *N*_a_ ≤ 10 and *N*_a_ > 10). Figure 6(a) shows the VAFs of Cu atoms in three groups and apparent damped oscillation behaviors observed with a little difference for the three groups of atoms. The difference is more obvious in the vibrational density of states (VDOS) Fourier-transformed from VAFs. As shown in the inset of Fig. 6(a), with the activated times increasing, the VDOS around 2 THz decreases, while those below 1.5 THz increases. This indicates that the atoms with higher activation probability participate more in the low-frequency modes, which are used to identify the “soft spots” in disordered solids where particles rearrangements or plastic events are initiated^43^. Thus, the atoms with higher activation probability in FUs are closely correlated with the “soft spots”. This is also consistent with the results that local structures involved in the low-frequency vibrations may have higher rate of structural transition^44^. Moreover, the more disordered regions in amorphous argon were found to be related to the excess of the low-frequency vibrations^45^. This may also imply that the atomic structures in FUs of metallic glasses could be more distorted.

The Lindemann parameter *δ*, which is a criterion of melting^43, 44^, was adopted to quantitatively characterize the fluidity of the FUs in MGs. *δ* is defined as, $\delta ={\mu }^{21/2}/{R}_{NN}$ where *μ* is the atom displacement in the time interval of 2 ns at 300 K, and *R*_NN_ is the average nearest neighbor distance and chosen as the position of first peak in radial distribution function. The lower panel in Fig. 6(b) shows the averaged Lindemann parameter *δ* of Cu atoms grouped with activated times. It is clearly shown that the averaged *δ* is monotonically increasing as activated times increase. Based on the Lindemann criterion, the melting of a crystalline solid and converting to liquid state is usually found to occur when *δ* reaches 0.07 or above^46, 47^. It is clearly shown that the *δ* value of the atoms with *N*_a _= 0 is below 0.07, while those of the atoms with *N*_a_ > 0 is above 0.07. We also doubled the simulation time scale to 4 ns to check its effect on the *δ* value, and the atom displacement was almost the same and the above results do not change. Therefore, the atoms with zero activation probability can be regarded as in solid state, while the atoms activated in dynamical tests are essentially in liquid state, especially for the atoms with much higher activation probability. In summary, FUs in MGs are indeed liquid-like.

The liquid-like feature of FUs in MGs can be clearly illustrated by the typical trajectories of atoms with different activation probabilities [see the inset in Fig. 6(b)]. For the atoms with *δ* < 0.07 in solid state, the motions of these atoms are mostly confined in the cages formed by their nearest neighbors and rattling. However, for the atoms in FUs with largest activation probability, their trajectories are much more broad and extensive, even far away from their initial positions. Therefore, the atoms with *δ* < 0.07 are indeed liquid-like. On the other hand, the liquid-like atoms in FUs tend to form clusters and distribute inhomogeneously in MGs as shown in the left lower inset in Fig. 2(b) and upper panel in Fig. 6(b), where the liquid-like FUs are enclosed by the solid-like elastic matrix, illustrating the heterogeneous structure and dynamics feature in MGs.

## Conclusions

In conclusions, based on statistical studies on the changes of the number of nearest neighbors of dynamical tests, we find the exact spatial location and activation probability of FUs in the initial structure of MG. We find that the FUs in MGs are loosely packed soft sites, liquid-like, participating more in the low-frequency vibrational modes and exhibiting relatively larger atomic mobility.

## References

[1] Greer AL (1995). Metallic glasses. Science.

[2] Wang WH (2009). Bulk metallic glasses with functional physical properties. Adv. Mater..

[3] Busch R, Schroers J, Wang WH (2007). Thermodynamics and kinetics of bulk metallic glass. MRS Bull..

[4] Greer AL, Ma E (2007). Bulk metallic glasses: at the cutting edge of metals research. MRS Bull..

[5] Spaepen FA (1977). Microscopic mechanism for steady state inhomogeneous flow in metallic glasses. Acta Metall. Mater..

[6] Argon AS (1979). Plastic deformation in metallic glasses. Acta Metall. Mater..

[7] Falk ML, Langer JS (1998). Dynamics of viscoplastic deformation in amorphous solids. Phys. Rev. E.

[8] Maloney CE, Lemaitre A (2006). Amorphous systems in athermal, quasistatic shear. Phys. Rev. E.

[9] Delogu F (2009). Effects of compression cycles on the atomic mobility in metallic glasses. Phys. Rev. B.

[10] Delogu F (2009). Atomistic simulation of local rearrangements in Ni_50_Zr_50_ metallic glasses subjected to compression cycles. Intermetallics.

[11] Delogu F (2011). Thermally-activated atomic rearrangements in elastically deformed metallic glasses. Mater. Chem. Phys..

[12] Delogu F (2012). Thermal activation of atomic rearrangements in elastically deformed Ni_50_Zr_50_ metallic glasses. Mater. Chem. Phys..

[13] Cheng YQ, Ma E (2011). Atomic-level structure and structure–property relationship in metallic glasses. Prog. Mater. Sci..

[14] Liu YH (2011). Characterization of nanoscale mechanical heterogeneity in a metallic glass by dynamic force microscopy. Phys. Rev. Lett..

[15] Peng HL, Li MZ, Wang WH (2011). Structural signature of plastic deformation in metallic glasses. Phys. Rev. Lett..

[16] Peng HL (2012). Characterization of mechanical heterogeneity in amorphous solids. J. Appl. Phys..

[17] Johnson WL, Samwer K (2005). A Universal Criterion for Plastic Yielding of Metallic Glasses with a (*T*/*T*_*g*_)^2/3^ Temperature Dependence. Phys. Rev. Lett..

[18] Ichitsubo T (2005). Microstructure of fragile metallic glasses inferred from ultrasound-accelerated crystallization in Pd-based metallic glasses. Phys. Rev. Lett..

[19] Liu YH, Wang G, Wang RJ, Pan MX, Wang WH (2007). Super plastic bulk metallic glasses at room temperature. Science.

[20] Li MZ, Wang CZ, Hao SG, Kramer MJ, Ho KM (2009). Structural heterogeneity and medium-range order in Zr_x_Cu_100-x_ metallic glasses. Phys. Rev. B.

[21] Falk ML, Langer JS (2011). Deformation and failure of amorphous, solidlike materials. Ann. Rev. Condens. Matter Phys..

[22] Dmowski W, Iwashita T, Chuang CP, Almer J, Egami T (2010). Elastic heterogeneity in metallic glasses. Phys. Rev. Lett..

[23] Wagner H (2011). Local elastic properties of a metallic glass. Nat. Mater..

[24] Ye JC, Lu J, Liu CT, Wang Q, Yang Y (2010). Atomistic free-volume zones and inelastic deformation of metallic glasses. Nat. Mater..

[25] Huo LS, Zeng JF, Wang WH, Liu CT, Yang Y (2013). The dependence of shear modulus on dynamic relaxation and evolution of local structural heterogeneity in a metallic glass. Acta Mater..

[26] Wang Z, Wen P, Huo LS, Bai HY, Wang WH (2012). Signature of viscous flow units in apparent elastic regime of metallic glasses. Appl. Phys. Lett..

[27] Mendelev MI, Sordelet DJ, Kramer MJ (2007). Using atomistic computer simulations to analyze x-ray diffraction data from metallic glasses. J. Appl. Phys..

[28] Jiao W (2013). Evolution of structural and dynamic heterogeneities and activation energy distribution of deformation units in metallic glass. Appl. Phys. Lett..

[29] Tomida T, Egami T (1993). Molecular-dynamics study of structural anisotropy and anelasticity in metallic glasses. Phys. Rev. B.

[30] Zhang Y, Mattern N, Eckert J (2012). Study of structural anisotropy in Cu_50_Zr_45_Al_5_ metallic glass under uniaxial compression by molecular dynamics simulations. Intermetallics.

[31] Conrad JC, Dhillon PP, Weeks ER, Reichman DR, Weitz DA (2006). Contribution of slow clusters to the bulk elasticity near the colloidal glass transition. Phys. Rev. Lett..

[32] Widmer-Cooper A, Harrowell P (2006). Predicting the long-time dynamic heterogeneity in a supercooled liquid on the basis of short-time heterogeneities. Phys. Rev. Lett..

[33] Liu ST, Wang Z, Peng HL, Yu HB, Wang WH (2012). The activation energy and volume of flow units of metallic glasses. Scr. Mater..

[34] Liu ST, Jiao W, Sun BA, Wang WH (2013). A quasi-phase perspective on flow units of glass transition and plastic flow in metallic glasses. J. Non-Cryst. Solids.

[35] Zhu ZG (2013). Characterization of flow units in metallic glass through structural relaxations. J. Appl. Phys..

[36] Xue RJ (2013). Characterization of flow units in metallic glass through density variation. J. Appl. Phys..

[37] Cheng YQ, Cao AJ, Sheng HW, Ma E (2008). Local order influences initiation of plastic flow in metallic glass: Effects of alloy composition and sample cooling history. Acta Mater..

[38] Lekka CE, Ibenskas A, Yavari AR, Evangelakis GA (2007). Tensile deformation accommodation in microscopic metallic glasses via subnanocluster reconstructions. Appl. Phys. Lett..

[39] Schall P, Weitz DA, Spaepen F (2007). Structural rearrangements that govern flow in colloidal glasses. Science.

[40] Wang WH (2011). Correlation between relaxations and plastic deformation, and elastic model of flow in metallic glasses and glass-forming liquids. J. Appl. Phys..

[41] Shi Y, Falk ML (2005). Strain localization and percolation of stable structure in amorphous solids. Phys. Rev. Lett..

[42] Harmon JS, Demetriou MD, Johnson WL, Samwer K (2007). Anelastic to plastic transition in metallic glass-forming liquids. Phys. Rev. Lett..

[43] Manning ML, Liu AJ (2011). Vibrational modes identify soft spots in a sheared disordered packing. Phys. Rev. Lett..

[44] Ashton DJ, Garrahan JP (2009). Relationship between vibrations and dynamical heterogeneity in a model glass former: Extended soft modes but local relaxation. Eur. Phys. J. E Soft Matter..

[45] Luchnikov VA, Medvedev NN, Naberukhin YI, Novikov VN (1995). Inhomogeneity of the spatial distribution of vibrational modes in a computer model of amorphous argon. Phys. Rev. B.

[46] Lindemann FA (1910). The calculation of molecular Eigen-frequencies. Phys. Z.

[47] Qiu W (2014). Part-crystalline part-liquid state and rattling-like thermal damping in materials with chemical-bond hierarchy. Proc. Natl. Acad. Sci..

